# Pharmacovigilance analysis of the Vigibase on neonatal withdrawal syndrome following in utero exposure to antidepressants

**DOI:** 10.1192/j.eurpsy.2023.278

**Published:** 2023-07-19

**Authors:** C. Gastaldon, E. Arzenton, E. Raschi, O. Spigset, D. Papola, G. Ostuzzi, U. Moretti, G. Trifirò, C. Barbui, G. Schoretsanitis

**Affiliations:** 1 WHO Collaborating Centre for Research and Training in Mental Health and Service Evaluation Department of Neuroscience, Biomedicine and Movement Sciences Section of Psychiatry; 2 Section of Pharmacology, Department of Diagnostics and Public Health, University of Verona, Verona; 3 Pharmacology Unit, Department of Medical and Surgical Sciences, University of Bologna, Bologna, Italy; 4 Department of Clinical Pharmacology, St Olav University Hospital, Trondheim, Norway and Department of Clinical and Molecular Medicine, Norwegian University of Science and Technology, Trondheim, Norway; 5 The Zucker Hillside Hospital, Department of Psychiatry, and Department of Psychiatry, Zucker School of Medicine, at Northwell/Hofstra, New York, United States

## Abstract

**Introduction:**

Evidence on neonatal withdrawal syndrome following antidepressant intrauterine exposure is limited, particularly for antidepressants other than selective serotonin reuptake inhibitors (SSRIs).

**Objectives:**

To ascertain whether maternal antidepressant treatment may be associated with withdrawal syndrome in neonates, investigating the comparative reporting between individual antidepressants and classes.

**Methods:**

We performed a case/non-case pharmacovigilance study, searching reports of withdrawal syndrome in newborns in the VigiBase, the WHO database of suspected adverse drug reactions. Disproportionality analysis was performed, estimating reporting odds ratio (ROR) and the Bayesian information component (IC). Antidepressants were compared to all other medications, to methadone, and within each class of antidepressants (SSRIs, tricyclics (TCA) and other antidepressants). Antidepressants were ranked in terms of clinical priority, based on a semiquantitative score.

**Results:**

We retrieved 406 reports of neonatal withdrawal syndrome in 379 neonates related to 15 antidepressants. Compared to all other drugs, disproportionate reporting was detected for antidepressants altogether (ROR: 6.18, 95%CI:5.45-7.01), for TCAs (10.55, 95%CI:8.02-13.88), other antidepressants (ROR: 5.90, 95%CI:4.74-7.36) and SSRIs (ROR: 4.68, 95%CI:4.04-5.42). All antidepressants showed a significant disproportionality, apart from bupropion (figure 1). We did not find any disproportionate reporting for any antidepressant compared to methadone. The clinical priority ranking showed moderate clinical priority for all antidepressants, with the exception four, that had a weak one (figure 1). Most frequently reported symptoms were respiratory symptoms (n=106), irritability/agitation (n=75), tremor (n=52) and feeding problems (n=40).

**Image:**

**Figure:**
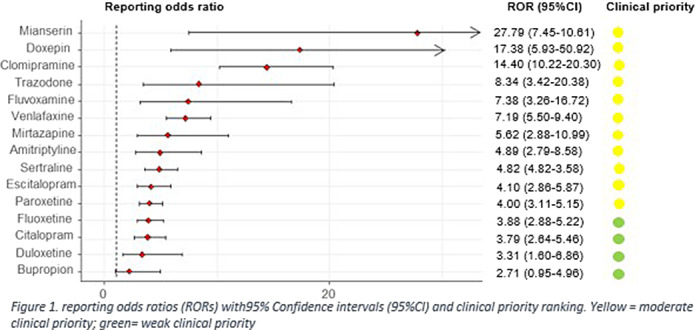

**Conclusions:**

Exposure to antidepressants in utero is associated with moderate signals of disproportionate reporting for neonatal withdrawal syndrome for most antidepressants. Clinicians should pay extra attention to neonates with antidepressant-treated mothers.

**Disclosure of Interest:**

C. Gastaldon: None Declared, E. Arzenton : None Declared, E. Raschi: None Declared, O. Spigset: None Declared, D. Papola: None Declared, G. Ostuzzi: None Declared, U. Moretti: None Declared, G. Trifirò Grant / Research support from: he was the scientific director of a Master program on pharmacovigilance, pharmacoepidemiology and real-world evidence which has received non-conditional grant from various pharmaceutical companies; he coordinated a pharmacoepidemiology team at the University of Messina until Oct 2020, which has received funding for conducting observational studies from various pharmaceutical companies (Boehringer Ingelheim, Daichii Sankyo, PTC Pharmaceuticals). He is also scientific coordinator of the academic spin-off “INSPIRE srl” which has received funding for conducting observational studies from contract research organizations (RTI Health Solutions, Pharmo Institute N.V.)., Consultant of: has served in the last three years on advisory boards/seminars funded by SANOFI, Eli Lilly, AstraZeneca, Abbvie, Servier, Mylan, Gilead, Amgen, Speakers bureau of: has served in the last three years on advisory boards/seminars funded by SANOFI, Eli Lilly, AstraZeneca, Abbvie, Servier, Mylan, Gilead, Amgen, C. Barbui: None Declared, G. Schoretsanitis Consultant of: Dr. Schoretsanitis has served as a consultant for HLS Therapeutics and Thermo Fisher Scientific.

